# HIF-1 Regulates Iron Homeostasis in *Caenorhabditis elegans* by Activation and Inhibition of Genes Involved in Iron Uptake and Storage

**DOI:** 10.1371/journal.pgen.1002394

**Published:** 2011-12-15

**Authors:** Steven Joshua Romney, Ben S. Newman, Colin Thacker, Elizabeth A. Leibold

**Affiliations:** 1 Department of Medicine, University of Utah, Salt Lake City, Utah, United States of America; 2 University of Washington, Seattle, Washington, United States of America; 3 Department of Biology, University of Utah, Salt Lake City, Utah, United States of America; 4 Department of Oncological Sciences, University of Utah, Salt Lake City, Utah, United States of America; Uuniversity of California San Diego, United States of America

## Abstract

*Caenorhabditis elegans ftn-1* and *ftn-2*, which encode the iron-storage protein ferritin, are transcriptionally inhibited during iron deficiency in intestine. Intestinal specific transcription is dependent on binding of ELT-2 to GATA binding sites in an iron-dependent enhancer (IDE) located in *ftn-1* and *ftn-2* promoters, but the mechanism for iron regulation is unknown. Here, we identify HIF-1 (hypoxia-inducible factor -1) as a negative regulator of ferritin transcription. HIF-1 binds to hypoxia-response elements (HREs) in the IDE in vitro and in vivo. Depletion of *hif-1* by RNA interference blocks transcriptional inhibition of *ftn-1* and *ftn-2* reporters, and *ftn-1* and *ftn-2* mRNAs are not regulated in a *hif-1* null strain during iron deficiency. An IDE is also present in *smf-3* encoding a protein homologous to mammalian divalent metal transporter-1. Unlike the *ftn-1* IDE, the *smf-3* IDE is required for HIF-1–dependent transcriptional activation of *smf-3* during iron deficiency. We show that *hif-1* null worms grown under iron limiting conditions are developmentally delayed and that depletion of FTN-1 and FTN-2 rescues this phenotype. These data show that HIF-1 regulates intestinal iron homeostasis during iron deficiency by activating and inhibiting genes involved in iron uptake and storage.

## Author Summary

Due to its presence in proteins involved in hemoglobin synthesis, DNA synthesis, and mitochondrial respiration, eukaryotic cells require iron for survival. Excess iron can lead to oxidative damage, while iron deficiency reduces cell growth and causes cell death. Dysregulation of iron homeostasis in humans caused by iron deficiency or excess leads to anemia, diabetes, and neurodegenerative disorders. All organisms have thus developed mechanisms to sense, acquire, and store iron. We use *Caenorhabditis elegans* as a model organism to study mechanisms of iron regulation. Our previous studies show that the iron-storage protein ferritin (FTN-1, FTN-2) is transcriptionally inhibited in intestine during iron deficiency, but the mechanisms regulating iron regulation are not known. Here, we find that hypoxia-inducible factor 1 (HIF-1) transcriptionally inhibits *ftn-1* and *ftn-2* during iron deficiency. We also show that HIF-1 activates the iron uptake gene *smf-3*. Transcriptional activation and inhibition by HIF-1 is dependent on an iron enhancer in the promoters of these genes. HIF-1 is a known transcriptional activator, but its role in transcriptional inhibition is not well understood. Our data show that HIF-1 regulates iron homeostasis by activating and inhibiting iron uptake and storage genes, and they provide insight into HIF-1 transcriptional inhibition.

## Introduction

Iron is essential due to its presence in proteins involved in DNA synthesis, mitochondrial respiration and oxygen transport. Regulation of cellular iron content is crucial: excess cellular iron catalyzes the generation of reactive oxygen species that damage DNA and proteins, while cellular iron deficiency causes cell cycle arrest and cell death. Dysregulation of iron homeostasis caused by iron deficiency or iron excess leads to hematological, neurodegenerative and metabolic diseases in humans. Iron must therefore be maintained within a narrow range to avoid the adverse consequences of iron depletion or excess.

Maintaining iron content within this physiological range requires precise mechanisms for regulating its uptake, storage and export (for reviews, see [1], [2]). In mammals, dietary non-heme Fe^3+^ is reduced by membrane bound ferric reductases (e.g. duodenal cytochrome B or DCYTB) before transport across the enterocyte apical membrane by divalent metal transporter-1 (DMT1, also known as NRAMP2, SLC11a2 and DCT1) [3]. Cytosolic iron is either transported across the basolateral membrane into the circulation by ferroportin or sequestered in ferritin in a form unable to catalyze free radical formation [4], [5]. Iron export by ferroportin is dependent on oxidation to Fe^3+^ by membrane and soluble multicopper oxidases where it is incorporated into transferrin for delivery to tissues. When body iron stores are high, cytosolic iron is not exported into blood, and is instead sequestered into ferritin [5], . Iron in ferritin is lost by sloughing of enterocytes into the intestinal lumen.

Mammalian intestinal iron transport increases during iron deficiency due to hypoxia-inducible factor-2α (HIF-2α) mediated expression of DMT1 and DCYTB [7], [8]. HIFs (HIF-1 and HIF-2) are key regulators of cellular and systemic oxygen homeostasis (for reviews, see [9], [10]). HIF transcription factors consist of an oxygen-regulated α subunit (HIF-1α, HIF-2α and a constitutively expressed β subunit (HIF-1β, also known as aryl hydrocarbon nuclear translocator or ARNT). In the presence of iron and oxygen, HIF-α subunits are hydroxylated by iron- and oxygen-dependent prolyl hydroxylases (PHDs) and are targeted for proteasomal degradation by the von Hippel-Lindau (VHL) E3 ubiquitin ligase. During hypoxia or iron deficiency, PHDs are inactivated, allowing HIF-1α /HIF-2β to accumulate. HIF-1α/HIF-2α dimerizes with HIF-β and binds to HREs in target genes to increase transcription. HIF regulates genes in diverse pathways including erythropoiesis, iron homeostasis, glucose metabolism, angiogenesis and cell survival (for reviews, see [9]–[11] ).

Oxygen and iron homeostasis pathways are conserved in *Caenorhabditis elegans*. The HIF-1 pathway in *C. elegans* consists of *hif-1*, *aha-1*, *vhl-1* and *egl-9*, which are orthologous to genes encoding mammalian HIF-1α, HIF-1β, VHL and PHD [12]–[14]. *C. elegans* express a single *hif* gene, which encodes a protein homologous to vertebrate HIF-1α and HIF-2α [12]. *C. elegans* HIF-1 regulates target genes involved in metabolism, extracellular remodeling [15], nervous system development [16], oxygen-dependent behavior [17] and modulation of life span [18]. *hif-1* mutant animals display increased embryonic and larval lethality in oxygen concentrations less than 1%, demonstrating the importance of HIF-1 for survival during hypoxia [13], [19].

*C. elegans* express genes homologous to ferritin (*ftn-1* and *ftn-2*), DMT1 (*smf-1*, *smf-2* and *smf-3*) and ferroportin (*fpn-1.1*, *fpn-1.2* and *fpn-1.3*). Vertebrate ferritin is a mixture of 24 light-(L) and heavy-(H) subunits that form a shell that can accommodate up to 4500 iron atoms. The H-subunits exhibit ferroxidase activity and facilitate the oxidation of iron, whereas the L-subunits function with the H-subunits in iron nucleation [20], [21]. *C. elegans* FTN-1 and FTN-2 display greater homology to the human H-subunit (55% and 60%) than to the L-subunit (46% and 50%), and notably both proteins contain ferroxidase active-site residues. *ftn-1* and *ftn-2* genes are transcriptionally repressed during iron deficiency, which is dependent on an iron-dependent enhancer (IDE) located in the *ftn-1* and *ftn-2* promoters [22]–[24]. The IDE contains two GATA binding sites for the intestinal specific ELT2 transcription factor that regulates basal *ftn-1* and *ftn-2* transcription, but the mechanism regulating iron-dependent transcriptional repression is unknown. Unlike *ftn-1* and *ftn-2*, vertebrate ferritin-H and -L subunit mRNAs are translationally repressed by iron-regulatory proteins 1 and 2 (IRP1 and IRP2) during iron deficiency (for reviews, see [25]–[27]). SMF-1, SMF-2 and SMF-3 display 55–58% amino acid identity with mammalian DMT1 and are involved in Mn^2+^ uptake and sensing, but the role of these transporters in iron uptake is not well understood [28]–[30]. The function of ferroportin homologs FPN1.1, FPN-1.2 and FPN-1.3 in iron homeostasis has not been reported.

Here, we show that HIF-1 activates *smf-3* transcription and inhibits *ftn-1* and *ftn-2* transcription during iron deficiency. Transcriptional activation of *smf-3* and repression of *ftn-1* and *ftn-2* is dependent on IDEs in their promoters that are similar but not identical. These studies show that HIF-1 is a key regulator of intestinal iron uptake and storage during iron deficiency in *C. elegans*.

## Results

### HIF-1 inhibits iron-dependent transcription of *ftn-1* and *ftn-2*

**Figure 1:**
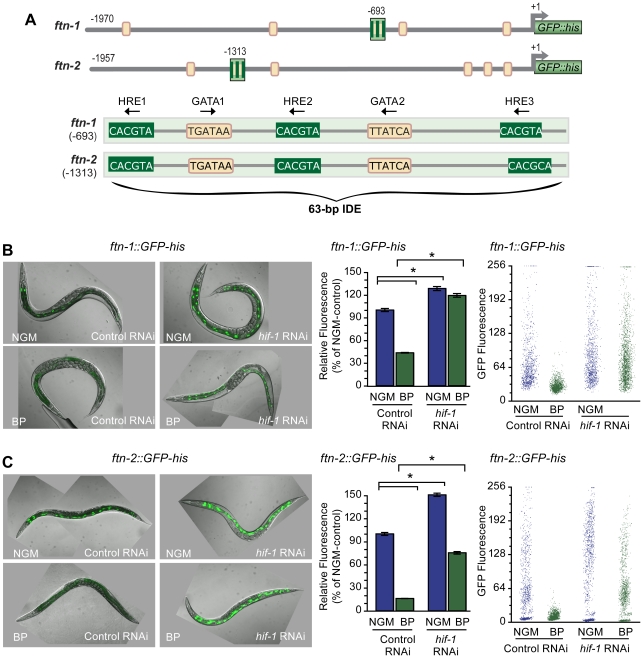
HIF-1 is required for *ftn-1* and *ftn-2* transcriptional repression during iron deficiency. (A) Conserved sequences in the 5′ promoter regions of *ftn-1* and *ftn-2*. *Large stripped boxes*, IDE; *small boxes*, GATA binding motifs. The IDE is enlarged showing HRE1, HRE2 and HRE3 and GATA1 and GATA2 sequences. Arrows indicate orientation of motifs. (B and C) Expression of *ftn-1::GFP-his* and *ftn-2::GFP-his* reporters in L4 larvae fed control (empty vector L4440) RNAi or *hif-1* RNAi after culture on NGM or NGM-BP for 16 h. Quantification of GFP expression in (B and C) was carried out using the COPAS Biosort. Dot plots of sorted worms are shown where each dot represents one worm. Data are mean GFP fluorescence ± SEM reported as a percentage of worms fed control RNAi on NGM (n = 1000 worms per treatment), *p<0.0001.

Previous studies showed that *ftn-1* and *ftn-2* transcription is activated by iron and inhibited by iron chelators [22]–[24]. Transcriptional regulation is mediated by the IDE located in the promoters of *ftn-1* and *ftn-2* genes (Figure 1A). The IDE contains two WGATAR sequences that are binding sites for the intestinal specific ELT2 GATA transcription factor. Mutation of either of the WGATAR sequences abolished expression of an *ftn-1::GFP-his* reporter showing that ELT-2 is required for *ftn-1* transcription under iron sufficient conditions [24]. The IDE also contains three canonical HREs (TACGTG) in the reverse orientation that have been identified in *hif-1* target genes [15], [31], suggesting a role for HIF-1 in iron-dependent *ftn-1* and *ftn-2* regulation.

To test this model, *hif-1* RNAi was used to deplete HIF-1 in worms carrying an *ftn-1::GFP-his* or an *ftn-2::GFP-his* reporter. GFP expression was quantified using the COPAS Biosort after growth in NGM or NGM supplemented with the membrane permeable Fe^2+^ chelator, 2,2′-dipyridyl (NGM-BP) [32]. These reporters contain 1.9 kb of *ftn-1* or *ftn-2* promoter sequences, including the IDE, fused to the initiator ATG of nuclear-localized GFP-histone [24]. BP reduces expression of *ftn-1::GFP-his* and *ftn-2::GFP-his* in worms fed control (empty vector L4400) RNAi by 60% and 80%, respectively, compared to worms grown on NGM (Figure 1B and 1C). By contrast, the BP- induced reduction in GFP expression is blocked by *hif-1* RNAi. Furthermore, *hif-1* RNAi increases GFP expression in worms grown on NGM, indicating that HIF-1 is expressed under normal growth conditions, and is capable of inhibiting *ftn-1* and *ftn-2* transcription.

**Figure 2:**
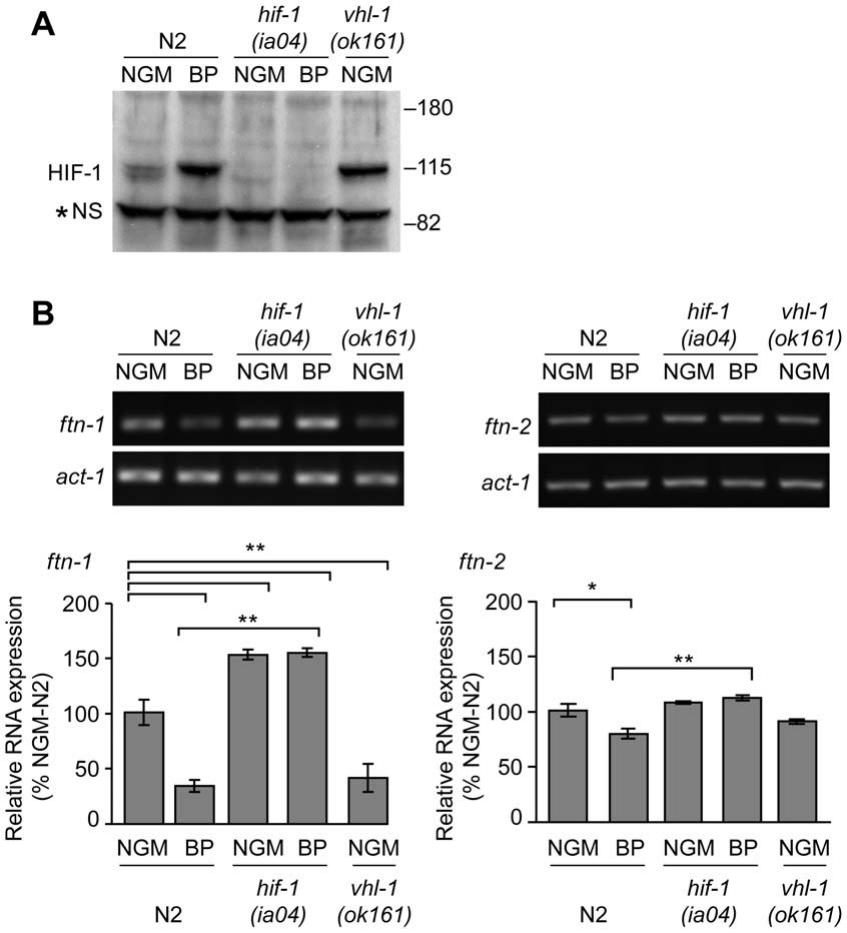
Endogenous *ftn-1* and *ftn-2* mRNA expression is regulated by HIF-1. (A) N2 wildtype, *hif-1(ia04)* and *vhl-1(ok161)* strains were cultured on NGM or NGM-BP, and HIF-1 was detected in extracts by western blots using *Ce*HIF-1 antibodies. *NS, non-specific band. (B) RNA was isolated from the strains in (A) and *ftn-1, ftn-2* and actin (*act-1*) mRNAs were quantified by RT-PCR. Relative changes in *ftn-1* and *ftn-2* mRNAs are expressed as a percentage of N2 wildtype animals on NGM after normalization to *act-1* mRNA. Five (*ftn-1*) and three (*ftn-2*) independent experiments were performed and the data are reported as means ± SEM, *p<0.05, **p<0.01.

We next determined whether endogenous *ftn-1* and *ftn-2* mRNAs are regulated by HIF-1. *ftn-1* and *ftn-2* mRNAs were measured in N2 wildtype animals cultured on NGM or NGM-BP and in strains carrying the loss-of function mutations in *hif-1*(*ia04*) and *vhl-1*(*ok161*). *vhl-1(ok161)* mutant animals lack VHL required for HIF-1 ubiquitination and proteasomal degradation, leading to constitutive expression of HIF-1 [14], [31], [33]. Western blots confirm the absence of HIF-1 in *hif-1(ia04*) mutant animals and increased HIF-1 levels in *vhl-1*(*ok161*) mutant and in N2 wildtype animals cultured in NGM-BP (Figure 2A). BP reduces *ftn-1* and *ftn-2* mRNA levels 75% and 20%, respectively, compared to untreated N2 wildtype animals. This is consistent with our previous studies showing that *ftn-1* is more sensitive to iron chelators as compared to *ftn-2* [22], [24]. By contrast, *ftn-1* and *ftn-2* mRNA levels are not reduced by BP in *hif-1(ia04*) mutant animals, and notably *hif-1(ia04)* animals cultured in NGM express higher amounts of *ftn-1* mRNA compared to N2 wildtype animals (Figure 2B). In *vhl-1(ok161)* mutant animals, *ftn-1* and *ftn-2* mRNAs are reduced to levels found in N2 wildtype animals cultured in NGM-BP. Taken together, these data show that *ftn-1* and *ftn-2* are transcriptionally inhibited by HIF-1 during iron deficiency.

### HIF-1 binds to the IDE in vitro and in vivo

**Figure 3:**
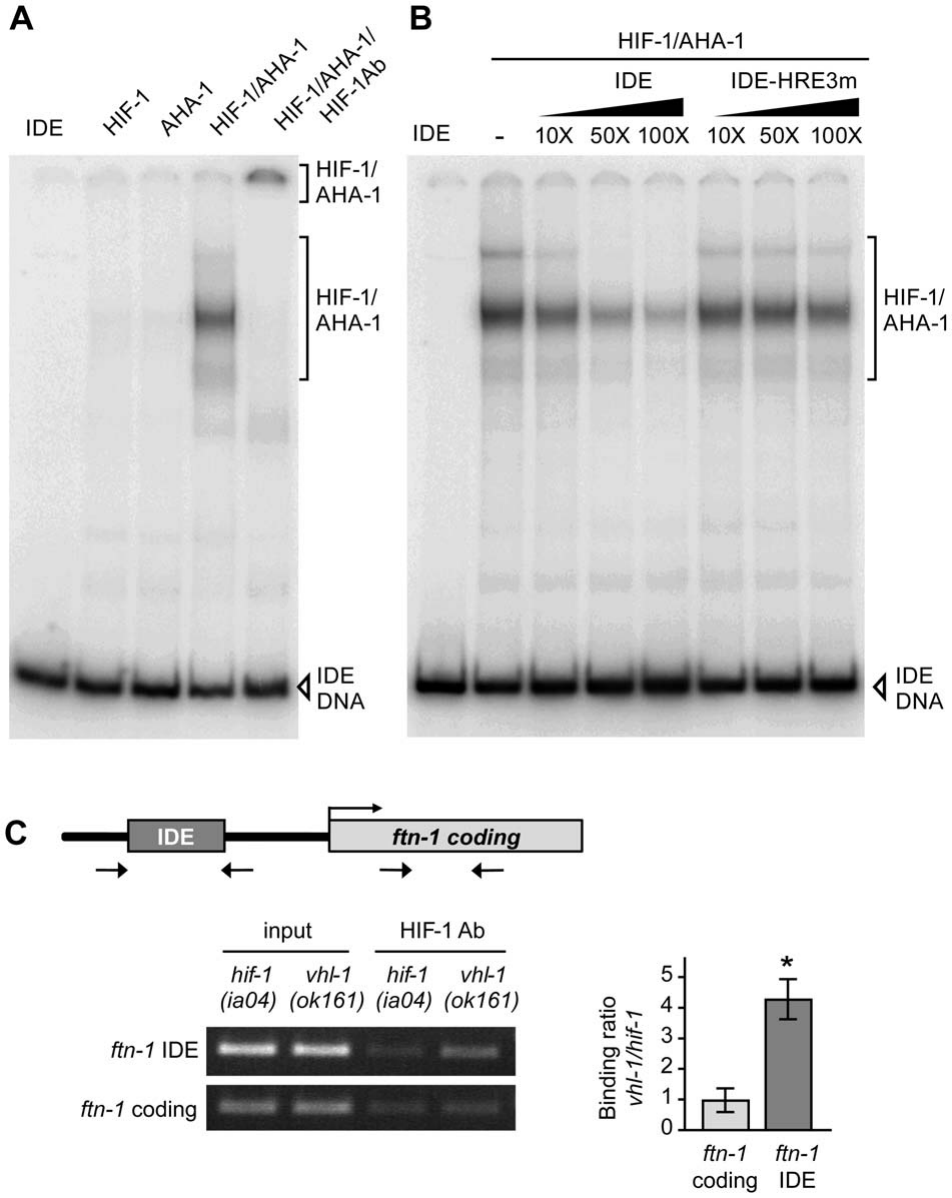
HIF-1 binds to the *ftn-1* IDE in vitro and in vivo. (A) HIF-1 and AHA-1 synthesized in reticulocyte lysates were incubated singly or together with a ^32^P-labeled *ftn-1* IDE probe. HIF-1/AHA-1/IDE complexes were analyzed by non-denaturing polyacrylamide gels. Free IDE probe and HIF-1/AHA-1/IDE complexes are indicated. HIF-1 antibody was added to the reaction (lane 5). (B) ^32^P-labeled IDE probe was incubated with HIF-1/AHA-1 with increasing molar amounts of unlabeled wildtype IDE or mutant IDE-HRE3m. (C) Diagram of the location of the IDE in the *ftn-1* promoter and the location of the primers used for amplification. ChIP was performed with chromatin isolated from a mixed-staged population of *vhl-1(ok161)* animals constitutively expressing HIF-1 or *hif-1(iao4)* animals. Chromatin was immunoprecipitated with *Ce*HIF-1 antibody and bound DNA sequences were analyzed by qPCR using IDE or coding region primers. Chromatin extract (*input*) was amplified to determine the total amount of genomic DNA prior to immunoprecipitation. The binding of HIF-1 to the IDE is expressed as a ratio of binding in *vhl-1(ok161)* versus *hif-1(ia04)* ± SEM, *p = 0.019. A representative gel of three independent experiments is shown.

Electrophoretic mobility shift assays were used to determine whether HIF-1 binds to the HREs in the IDE. Radiolabeled wildtype IDE or an IDE containing mutations in the three HRE sites (HRE3m) was incubated with reticulocyte lysate-synthesized HIF-1 and AHA-1 (Figure 3A). Complex formation is only observed when HIF-1 and AHA-1 are present together in the reaction with wildtype IDE (Figure 3A). Addition of HIF-1 antibody to the reaction led to the formation of a slower migrating HIF-1/AHA-IDE complex (Figure 3A, lane 5). Formation of HIF-1/AHA-1-IDE complexes is competed away by unlabeled wildtype IDE but not by unlabeled HRE3m IDE, showing that HIF-1 specifically binds to the HREs (Figure 3B).

To determine whether the IDE is a direct HIF-1 target *in vivo*, ChIP was performed on chromatin isolated from *vhl-1*(*ok161*) and *hif-1(ia04)* mutant animals. The binding of HIF-1 to the IDE or to the *ftn-1* coding region used as a negative control was determined by ChIP using HIF-1 antibody. IDE DNA was enriched 4-fold in *vhl-1*(*ok161*) immunoprecipitates as compared to *hif-1(ia04)* immunoprecipitates (Figure 3C). These studies indicate that HIF-1 binds to HREs in vitro and occupies the *ftn-1* IDE in vivo.

### The DMT1 ortholog SMF-3 is regulated by HIF-1 during iron deficiency

**Figure 4:**
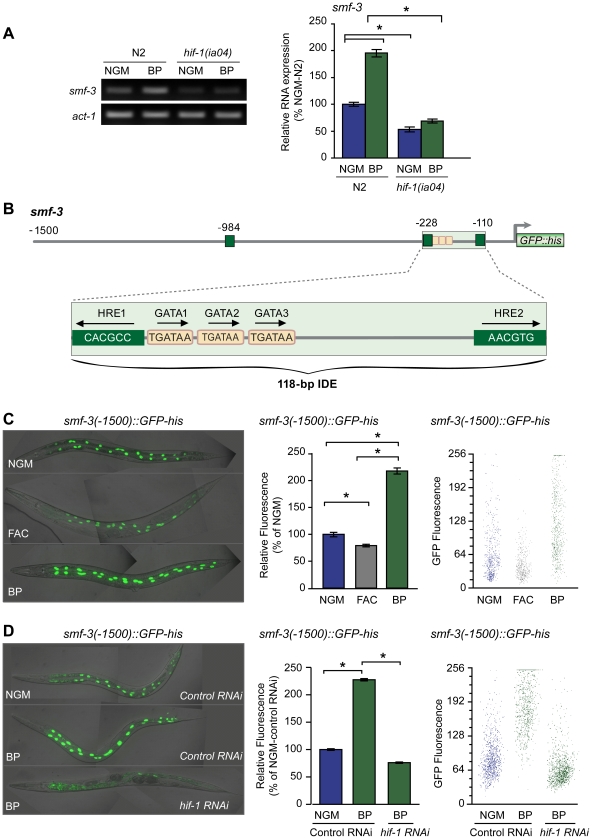
Activation of *smf-3* during iron deficiency is dependent on an IDE in the *smf-3* promoter. (A) *smf-3* mRNA was quantified by RT-PCR in RNA isolated from N2 wildtype and *hif-1(ia04)* mutant strains cultured in NGM or NGM-BP for 16 h. Relative changes in *smf-3* mRNA are expressed as a percentage of N2 wildtype animals cultured on NGM after normalization to *act-1* mRNA. Five independent experiments were performed and the data are reported as means ± SEM, *p<0.0015. (B) Schematic diagram of the 5′ promoter region of the *smf-3* gene. *Small boxes*, GATA binding motifs; *large boxes*, HREs. The IDE is enlarged showing HRE1, HRE2 and HRE3 and GATA1 and GATA2 sequences. Arrows indicate motif orientation. (C) Expression *smf-3(-1500)::GFP-his* containing 1.5 kb of promoter sequences in worms after culture in NGM, NGM-BP or NGM supplemented with FAC for 18 h. (D) Expression of *smf-3(-1500)::GFP-his* reporter in L4 animals fed control RNAi or *hif-1* RNAi after culture in NGM or NGM-BP for 16 h. GFP fluorescence was quantified in (C and D) using the COPAS Biosort as described in Figure 1. Data are mean GFP fluorescence ±SEM as a percentage of untreated worms grown in NGM for (C) or as a percentage of worms fed control RNAi on NGM for (D), n = 1000 worms per treatment, *p<0.001.

The activation of DMT1 by HIF-2α in iron-deficient mice increases intestinal iron uptake [7], [8]. *C. elegans* express three DMT1 homologs, SMF-1, SMF-2 and SMF-3. SMF-1 and SMF-3 are expressed in the apical membrane in intestinal cells and are involved in Mn^2+^ uptake [28], [30], whereas SMF-2 is mainly expressed in pharyngeal epithelial cells [28]–[30]. To determine whether HIF-1 regulates *smf-1*, *smf-2* or *smf-3* expression during iron deficiency, their mRNA levels were quantified in N2 wildtype and *hif-1(ia04)* mutant animals cultured in NGM or NGM-BP. BP increases *smf-3* mRNA levels 2-fold as compared to untreated N2 wildtype animals, but has no effect on *smf-1* or *smf-2* mRNA levels (Figure 4A and data not shown). In *hif-1(ia04)* mutant animals, *smf-3* mRNA levels are reduced by 50% as compared to N2 wildtype animals, and are not increased by BP (Figure 4A).

Inspection of the 5′ upstream regulatory region of *smf-3* reveals a 118-nt element harboring three tandem GATA binding sites flanked by two HREs (Figure 4B). To determine whether this element functions as an iron enhancer, transgenic strains carrying a transcriptional reporter containing 1500 nt of *smf-3* promoter sequences fused to *GFP-his* were generated. GFP expression was quantified in these strains after growth on NGM, NGM-ferric ammonium citrate (NGM-FAC) or NGM-BP. FAC reduces GFP expression, whereas BP increases GFP expression as compared to untreated worms (Figure 4C). To show that the BP-induced increase in GFP expression is due to iron chelation, GFP expression was quantified after culture of worms on NGM-BP in the presence of an equimolar amount of FAC or MnCl_2_. BP plus FAC, but not BP plus MnCl_2_, reduces *smf-3(-1500)*::*GFP-his* expression (Figure S1). *hif-1* RNAi completely blocks BP-induced increased GFP expression (Figure 4D). Similarly, expression of a *smf-3(-1500)::GFP-his* reporter containing 250 nt of upstream sequences containing the IDE is increased by BP in control RNAi fed worms and is reduced by BP in *hif-1* RNAi fed worms (Figure S2). Taken together, these data indicate that *smf-3* is transcriptionally activated by HIF-1 during iron deficiency.

### Iron content is reduced in *smf-3(ok1035)* mutant animals

**Figure 5:**
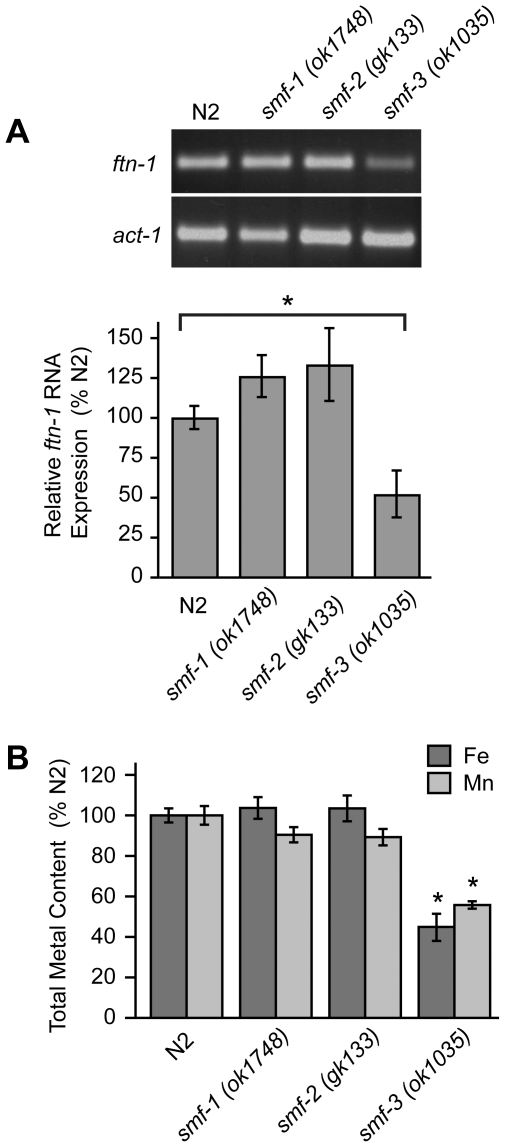
Total Fe content is reduced in *smf-3(1035)* mutant animals. (A) *ftn-1* mRNA was quantified by RT-PCR from RNA isolated from N2 wildtype and *smf-1(ok1748)*, *smf-2(gk133)* and *smf-3(ok1035)* strains. Relative changes in *ftn-1* mRNA are expressed as a percentage of N2 wildtype animals after normalization to *act-1* mRNA. Five independent experiments were performed and data are reported as means ± SEM, * *p*<0.05. (B) Total content of Fe and Mn in N2 wildtype and *smf* mutant animals determined by ICP spectroscopy is expressed as a percentage of N2 wildtype animals for each metal. At least three independent experiments were performed and the data are reported as means ± SEM, *p<0.01.

The localization of SMF-3 to the apical membrane of intestinal cells [28], [30] and its regulation by HIF-1 suggest a role for SMF-3 in intestinal iron uptake. If SMF-3 has a role in iron uptake, iron content might be expected to be reduced in *smf-3(ok1035)* mutant animals. We found that *ftn-1* mRNA levels, which are positively correlated with cellular iron levels (Figure 2), are reduced in *smf-3(ok1035)* mutants as compared to N2 wildtype animals, but not in *smf-1(ok1748)* or *smf-2(gk133)* mutant animals (Figure 5A). Quantification of metal content by inductively-coupled plasma spectroscopy (ICP) shows that total iron content in *smf-3(ok1035)* mutant animals is 45% of N2 wildtype animals consistent with reduced *ftn-1* mRNA levels in these animals (Figure 5B). The total iron content in *smf-1(ok1748)* and *smf-2(gk133)* mutant animals is not significantly different as compared to N2 wildtype animals. The total Mn content in *smf-3(ok1035)* mutant animals is 60% of N2 wildtype controls (Figure 5B) in agreement with SMF-3 as a regulator of Mn uptake [28], [30]. The Mn content in the *smf-1(ok1748*) and *smf-2(gk133)* strains tended to be lower as compared to N2 wildtype controls, but did not reach significance. These results are consistent with a role for SMF-3 in intestinal iron transport.

### Reduced viability of *hif-1(ia04)* mutant animals by iron deficiency is rescued by *ftn-1* and *ftn-2* RNAi

**Figure 6:**
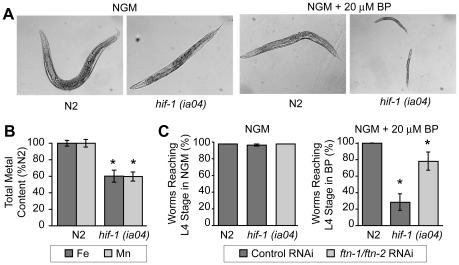
Delayed growth of *hif-1(ia04)* mutant animals during iron deficiency is rescued by *ftn-1* and *ftn-2* RNAi. (A) N2 wildtype and *hif-1(ia04)* mutant L1 stage animals were grown on NGM with or without 20 µM BP for 48 h. *hif-1(ia04)* mutant animals are developmentally delayed when grown on NGM-20 µM BP as compared to growth on NGM. (B) Total iron content in N2 wildtype and *hif-1(ia04)* mutant animals determined by ICP is expressed as a percentage of N2 wildtype animals. Four independent experiments were performed and the data are reported as means ± SEM, *p<0.02. (C) N2 wildtype and *hif-1(ia04)* animals were fed control RNAi or *ftn-1* and *ftn-2* RNAi and cultured on NGM or NGM+20 µM BP. The data are expressed as the number of animals reaching L4 stage as a percentage of N2 wildtype animals. Three independent experiments were performed and the data are reported as means ± SEM, *p<0.05, *hif-1(ia04)* (control RNAi) versus *hif-1(ia04*) (*ftn-1/ftn-2* RNAi).

*hif-1(ia04)* mutant animals have no overt phenotype in normoxia, but display reduced viability in oxygen concentrations less than 1% [13], [34]. Similarly, we find that *hif-1(ia04)* mutant animals develop normally under normoxic conditions, but are developmentally delayed when cultured under iron deficient normoxic conditions (NGM plus 20 µM BP) as compared to N2 wildtype animals (Figure 6A). As total iron content is reduced in *smf-3(ok1035)* mutant animals and *smf-3* expression is reduced in *hif-1(ia04)* animals, these data suggest that total iron content might also be reduced in *hif-1(ia04)* animals. ICP analyses show that the total iron content in *hif-1(ia04)* mutant animals is 60% of N2 wildtype animals (Figure 6B). The total Mn content is also reduced in *hif-1(ia04)* mutant animals.

We next questioned whether the developmental delay observed in *hif-1(ia04)* mutant animals cultured in BP can be rescued by reducing FTN-1 and FTN-2 expression, which would lead to an increase in the cellular labile iron pool. This pool contains chelatable redox-active iron that constitutes less than 5% of total cellular iron [32], [35]. The modulation of ferritin levels is one mechanism for regulating the labile iron pool in mammalian cells: ferritin overexpression reduces the iron pool, while ferritin depletion increases this pool [36]–[38]. We depleted *ftn-1* and *ftn-2* by RNAi in N2 wildtype and in *hif-1(ia04)* mutant animals cultured on NGM or NGM plus 20 µM BP, and the number of worms reaching L4 stage was measured. *ftn-1/ftn-2* RNAi increases the number of *hif-1(ia04)* mutant animals reaching L4 stage from 28% in untreated animals to 78% in *ftn-1/ftn-2* RNAi-fed animals (Figure 6C). These data indicate that the developmental delay observed in *hif-1(ia04)* mutant animals during iron deficiency can be partially rescued by reducing FTN-1 and FTN-2 levels.

## Discussion

Previous studies showed that *ftn-1* and *ftn-2* transcription in intestine is activated by iron and inhibited by iron deficiency [22]–[24]. Transcriptional regulation is dependent on an IDE containing HIF-1 and GATA binding sites. The GATA factor ELT2 regulates basal intestinal *ftn-1* and *ftn-2* transcription during iron sufficiency, but how transcription is repressed by iron deficiency was not understood. Here, we identify HIF-1 as a negative regulator of *ftn-1* and *ftn-2* transcription during iron deficiency. We also show that *smf-3* is regulated by HIF-1 during iron deficiency, but unlike *ftn-1* and *ftn-2*, HIF-1 activates *smf-3* transcription. The activation of *smf-3* and inhibition of *ftn-1* and *ftn-2* by HIF-1 provides a mechanism to increase iron uptake and decrease iron storage during iron deficiency.

In mice, increased iron uptake by DMT1 during iron deficiency is mediated by HIF-2α [7], [8]. In contrast to the transcriptional regulation of *ftn-1* and *ftn-2* in *C. elegans*, vertebrate ferritin H- and -L subunit mRNAs are translationally repressed during iron deficiency by the binding of IRP1 and IRP2 to an iron-responsive element (IRE) in ferritin mRNAs [26]. When cellular iron levels increase, IRP1 is converted into a cytosolic aconitase concomitant with loss of RNA-binding activity and IRP2 is degraded, which leads to increased ferritin translation. Although *C. elegans* express an IRP1 homolog (ACO-1), it lacks RNA-binding activity and functions solely as a cytosolic aconitase [22]. The regulation of iron homeostasis by *ftn-1*and *ftn-2* is essential as depletion of FTN-1 and FTN-2 rescues the growth delay observed in *hif-1* null worms grown under iron limiting conditions. Recent studies show that constitutive expression of ferritin-H and -L subunits in mice with intestinal specific deletion of IRP1 and IRP2 have reduced survival rates [39]. Depletion of the intestinal ferritin-H gene in mice leads to reduced iron sequestration in intestine and increased body iron stores [40]. Taken together, these studies show that the precise regulation of intestinal ferritin expression is essential for appropriate control of iron absorption.

The transcriptional inhibition of *ftn-1*and *ftn-2* genes by HIF-1 during iron deficiency was unexpected. HIF-1 is a potent transcriptional activator of hundreds of HRE target genes [15], [41], [42]. HIF has also been reported to function as a repressor, but the mechanism of transcriptional repression is not fully understood [43]–[48]. For some HIF-negatively regulated genes, transcriptional repression may be an indirect effect due to HIF activation of a transcriptional repressor [41], [42]. HIF-1 has also been shown to upregulate microRNA mir-120, which represses gene expression [49]. For other genes, direct binding of HIF to HREs in the promoters of negatively-regulated genes has been demonstrated using ChIP analysis [45]–[47]. These studies led to models whereby HIF-1 negatively regulates transcription by the recruitment of corepressors and histone modifying complexes or by competing with transcriptional activators for HRE binding.

One question is how HIF-1 mediates transcriptional activation and inhibition through the IDE. The *ftn-1*, *ftn-2* and *smf-3* IDEs differ in number, spacing and orientation of the GATA and HIF-1 binding sites. It is likely that architecture of the IDE dictates physical interactions of ELT-2, HIF-1, coactivators and other transcription factors to activate or repress transcription. Further studies are needed to determine how the structure of the IDE affects HIF-1 transcriptional responses.

HREs can be flanked by transcription factor binding sites, and it is the cooperation of HIF with these transcription factors that enhance transcription or direct cell specific expression [11]. The GATA binding sites flanking the HREs in *ftn-1*, *ftn-2* and *smf-3* are essential for intestinal expression because mutations of these sites or depletion of ELT-2 reduces intestinal expression of *ftn-1* and *smf-3* GFP transcriptional reporters [24] (preliminary data, SJR and EAL). Computational studies have shown that the majority of intestinal specific genes contain GATA binding sequences, leading to the notion that ELT2 is a global regulator of intestinal gene expression [50], [51]. Several studies have shown that other transcription factors cooperate with ELT-2 to modulate its function in response to nutritional or physiological signals [52]–[56].

**Figure 7:**
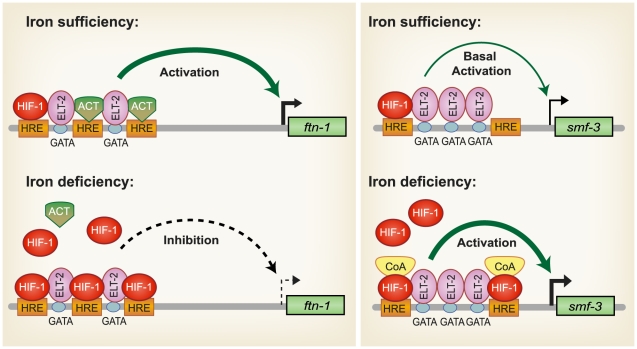
Model for HIF-1 iron-dependent activation and inhibition of intestinal iron uptake and storage. *Iron sufficiency*: ELT-2 binds to GATA binding sites located in the *ftn-1* and *ftn-2* IDEs. We propose that ELT- 2 cooperates with an unidentified transcriptional activator (ACT) that binds to the HREs and enhances transcription. *smf-3* is transcribed at basal levels during iron sufficiency. HIF-1 is expressed during normal growth conditions, but at low levels. *Iron deficiency*: HIF-1 is stabilized and dimerizes with AHA-1. HIF-1/AHA-1 (denoted by HIF-1) displaces the unidentified transcriptional activator binding to the *ftn-1* and *ftn-2* HREs and inhibits transcription. HIF-1/AHA-1 binds to the *smf-3* HREs, recruits coactivators and cooperates with ELT-2 to enhance *smf-3* transcription. Whether ELT-2 is bound to the *ftn-1* GATA sites during iron deficiency and to the *smf-3* GATA sites during iron sufficiency has not been determined.

Based on our findings, a model for HIF-1 regulation of intestinal iron homeostasis is proposed (Figure 7). During iron sufficiency, ELT-2 binds to GATA binding sites in the IDE to activate intestinal expression of *ftn-1* and *ftn-2*. Both GATA binding sites are required for *ftn-1* expression as deletion of either site abolished expression of an *ftn-1* GFP transcriptional reporter [24]. In addition, mutation of all three HREs in the *ftn-1* IDE abolished GFP reporter expression, suggesting that a transcriptional activator may bind to the HREs enhancing ELT-2 function [24]. This activator may be a member of the basic helix-loop-helix (bHLH/PAS) family or a bHLH transcriptional activator that binds to noncanonical E-box elements [57]–[59]. *smf-3* transcription is reduced during iron sufficiency due to decreased HIF-1α levels. Our data also show that a small amount of HIF-1 is expressed during normal growth conditions that can interact with HREs in *ftn-1* and *smf-3*. During iron deficiency, HIF-1 is stabilized and transported to the nucleus where it dimerizes with AHA-1. We propose that HIF-1/AHA-1 competes with a transcriptional activator for binding to the *ftn-1* and *ftn-2* HREs, inhibiting *ftn-1* and *ftn-2* transcription. HIF-1/AHA-1 binds to the *smf-3* HREs, recruits coactivators and cooperates with ELT-2 to activate transcription.

SMF-1, SMF-2 and SMF-3 have been characterized with regard to their role in Mn^2+^ homeostasis and sensitivity [28], [30]. SMF-1 and SMF-3 are localized to the apical intestinal epithelium consistent with a role in metal uptake [28], [30], whereas SMF-2 is primarily expressed in pharyngeal epithelium [28], [29]. SMF-1 and SMF-2 are also expressed in dopaminergic neurons where they mediate sensitivity of neurons to Mn and neurotoxins [29]. The high homology of SMF-3 with mammalian DMT1, its localization in the apical membrane of intestinal cells [28], [30], its regulation by iron and a reduction in total iron content in *smf-3(ok1035)* mutant animals indicate a role for SMF-3 is intestinal iron transport in *C. elegans*. The regulation of iron homeostasis by HIF-1 provides a mechanism to ensure that *C. elegans* maintain sufficient iron stores for growth and survival when iron is limiting.

## Materials and Methods

### *C. elegans* strains and culture

Strains were cultivated on nematode growth medium (NGM) agar plates seeded with *Escherichia coli* OP50 at 20–22°C. For iron chelation experiments, synchronized larvae were grown for 24 h on NGM plates then transferred to NGM plates supplemented with 100 uM 2,2′-dipyridyl (BP) for 18 h unless indicated. For iron experiments, larvae were grown for 18 h on NGM plates supplemented with 6.6 mg/ml ferric ammonium citrate (FAC). The pH of FAC-supplemented NGM agar was adjusted to pH 7.0.

The strains provided by *C. elegans* Genetics Center are: wild-type Bristol N2, *vhl-1* (*ok161*) [13], *hif-1* (*ia04*) [14], RB1491 *smf-1(ok1748)*, VC171 *smf-2(gk133)* and RB1074 *smf-3(ok1035)*. XA6900 *pha-1(e2123ts) III; qaEx1 [ftn-1::Δpes-10::GFP-his, pha-1+] and* XA6901 *lin-15(n765ts) X; qaEx2 [ftn-2::Δpes-10::GFP-his, lin-15+]* were previously described (Romney et al., 2008). Strains generated in this study are: XA6904 *pha-1 (e2123ts) III; qaEx04 [smf-3(-1500)::Δpes-10::GFP-his, pha-1+]* and XA6905 *pha-1(e2123ts) III; qaEx05 [smf-3(-250)::Δpes-10::GFP-his, pha-1+]*.

### Reporter constructs and transgenic lines

*smf-3(-1500)::GFP-his* and *smf-3(-250)::GFP-his* were generated by PCR amplification of sequences 1500 nt or 250 nt, respectively, upstream from the initiation ATG of *smf-3* (Y69A2AR.4) using primers containing *Sal*I and *Nhe*I restriction sites on the 5′ and 3′ termini, respectively. The PCR products were cloned into TOPO Zeroblunt (Invitrogen) followed by digestion and insertion into the *Sal*I and *Nhe*I sites of pAP.10. Transgenic strains were made according to standard microinjection procedures. Plasmids B696 (*smf-3(*-1500)*::GFP-his)* and B698 (*smf-3(-250)::GFP-his*) were each co-injected (20 ng/ul) with selection plasmid pBX-1(100 ng/ul). Transgenic worms were recovered after growth at 20–22°C.

### RNAi

RNAi clones against *ftn-1* (C54F6.14) and *ftn-2* (D1037.3) were from the ORFeome-based RNAi library [60] and *hif-1* (F38A6.3) was from the Ahringer feeding library [61]. Empty vector (L4400) was used as a control. Worms were grown on RNAi plates (NGM containing 1 mM IPTG and 50 ug/ml ampicillin) seeded with bacteria expressing dsRNA corresponding to *ftn-1*and *ftn-2*, *hif-1* or L4400.

### Rescue

Mixed-stage populations of N2 wildtype and *hif-1(ia04)* animals containing gravid adults were transferred to RNAi plates seeded with bacteria expressing *ftn-1* and *ftn-2* dsRNA or empty vector control (L4400) for 24 h at 20–22°C. Synchronized larval stage (L1) worms were obtained by treating RNAi fed gravid adults with alkaline hypochlorite and allowing eggs to hatch overnight in sterile S-basal media. L1 larvae were spotted on *ftn-1* and *ftn-2* or L4400 RNAi plates supplemented with or without 20 µM BP and incubated at 20–22°C for 48 h prior to scoring for larval stage using a Leica MZ9.5 stereomicroscope.

### *C. elegans* sorting

*ftn-1::GFP-his, ftn-2::GFP-his, smf-3(-1500)::GFP-his* and *smf-3(-250)::GFP-his* reporter lines were synchronized by treating gravid adults with alkaline hypochlorite followed by hatching eggs in S-basal medium (0.1 M NaCl, 0.05 M potassium phosphate, pH 6.0, 5 ug/ml cholesterol) overnight. Synchronized L1 worms were grown on control or *hif-1* RNAi plates for 32 h. Worms were then rinsed from the plates and washed in M9 buffer (22 mM KH_2_PO_4_, 42 mM Na_2_HPO_4_, 86 mM NaCl, 1 mM MgSO_4_) and spotted onto fresh 10 cm control or *hif-1* RNAi plates supplemented with 100 µM BP and grown for 18 h. Worms were rinsed from plates and washed with MT buffer (M9 buffer containing 0.1% Triton X-100) by repeated rounds of centrifugation until free of debris. Worms were analyzed using the COPAS Biosort (Union Biometrica, Somerville, MA) as described [24]. Prior to data acquisition, gating parameters were established by visualizing a sorted population by microscopy. The same gating parameters were used for all experimental conditions during GFP fluorescence acquisition and subsequent analysis. Data were analyzed using FCS Express Version 3.0 lite (De Novo Software, Ontario, Canada). Procedures for sorting non-RNAi-treated worms was the same as above except that synchronized L1 stage worms were grown on NGM plates seeded with OP50 for 32 h prior to being transferred to fresh NGM plates and NGM plates supplemented with either 100 uM BP, 100 uM FAC, 100 uM BP plus100 uM MnCl_2_ or 100 uM BP plus 100 uM FAC. Worms were grown for an additional 18 h prior to data acquisition as performed above.

### Microscopy

Images of GFP expression were captured using an Axio Imager (Carl Zeiss MicroImaging, Inc, Thornwood NY) outfitted with the Zeiss filter set 38HE (BP 470/40HE, dichroic FT 495 HE, BP 525/50 HE) and an AxioCam HRm camera (Carl Zeiss MicroImaging, Inc, Thornwood NY) using AxioVision software. Following acquisition, images were rotated, cropped and sized using Adobe Photoshop.

### Western blotting

Mixed-stage populations of N2, *hif-1(ia04)* and *vhl-1(ok161)* animals were transferred from NGM plates to fresh NGM and 100 uM BP supplemented plates for 16 hr prior to harvesting. Worms were collected, washed with ddH_2_O and resuspended in 200 µl lysis buffer (20 mM HEPES pH 7.5, 25 mM KCl, 0.5% NP-40). Worms were sonicated twice using a Misonix 3000 (8 pulses, output 3), centrifuged and the protein concentration of the clarified lysate was determined using Coomassie Plus protein assay reagent (Thermo). Samples (20 ug) were fractionated on a NuPAGE 4–12% Bis-Tris gel (Invitrogen) and western blotting was carried out using rabbit *Ce*HIF-1 antibody at (1∶5,000) [14] (a gift from Dr. Peter Ratcliffe, Oxford) followed by incubation with horseradish peroxidase-conjugated secondary antibodies. Proteins were visualized using Western Lighting Chemiluminescence Reagent Plus (PerkinElmer Life Sciences).

### RT–PCR

Synchronized L1 larvae were grown on NGM plates seeded with OP50 for 48 h prior to harvest. For iron chelation experiments, synchronized worms were transferred to new NGM plates or NGM plates supplemented with 100 uM BP after 32 h and harvested at 48 h. At harvest, worms were washed from plates and cleaned by repeated rounds of centrifugation and resuspension in ddH2O. Pelleted worms were resuspended in TRIZOL (Invitrogen) and total RNA was purified according to manufacturer's protocol. cDNA was synthesized using 1 ug of purified total RNA using SuperscriptIII Supermix for qRT-PCR (Invitrogen). Semiquantitative RT-PCR (sqRT-PCR) was performed using Recombinant Taq Polymerase (Invitrogen). PCR products were run on ethidium bromide stained 1.2% agarose gels. Images were captured on a FluorChem IS-8900 (Alpha Innotech) and analyzed using ImageQuaNT (Molecular Dynamics) and ImageJ. Primer sequences are shown in Table S1.

### Chromatin immunoprecipitation (ChIP)

ChIP assays were performed as previously described [62]. Mixed-stage *vhl-1* (*ok161*) and *hif-1* (*ia04*) worms were grown on two 150 mm NGM plates and harvested. Worm pellets were homogenized in crosslinking buffer (1% formaldehyde in PBS) and incubated for 20 min at room temperature. The reactions were quenched with glycine for 20 min and snap frozen. The frozen pellets were resuspended in HLB buffer (50 mM HEPES-KOH, pH 7.5, 150 mM NaCl, 1 mM EDTA, 0.1% sodium deoxycholate, 1% Triton-X100, 0.1% SDS, 1 mM PMSF) containing protease inhibitor cocktail (Calbiochem), sonicated and the supernatants were precleared using 30 ul salmon sperm DNA/protein A agarose beads. After centrifugation, 10% of each supernatant was kept as input, while the remaining supernatants were incubated with *Ce*HIF antibody (4 ul) overnight at 4°C. DNA-protein antibody complexes were incubated with Protein A agarose (30 ul slurry) for 2 h at 4°C, and the beads were then resuspended in proteinase K buffer (20 mg/ml proteinase K) and incubated at 45°C for 2 h. The samples were further incubated at 65°C overnight to reverse crosslinks. DNA was purified using a QIAquick PCR purification kit (Qiagen) according to manufacturer's protocol. Quantitative (q) PCR was then performed using Sybr Green mix (Invitrogen) run on an ABI 7000 Sequence Detection System and additionally visualized by 2% ethidium bromide stained agarose gels. Primers flanking the *ftn-1* IDE and control primers within the *ftn-1* coding region are shown in Table S1.

### Electrophoretic mobility shift assay (EMSA)

Wildtype IDE and IDE containing mutations in the three HRE binding sites (HRE3m) (CACGTAGC>ACATGCTA) were excised from TopoZero blunt (Invitrogen) with *EcoR*I and gel purified. Wildtype IDE was radiolabeled with 50 µCi of ^32^P[α-dATP] using Klenow DNA polymerase. HIF-1 and AHA-1 were synthesized in TNT SP6 Quick Coupled Transcription/Translation system (Promega) using *hif-1* (pSP64-HIF-1) and *aha-1* (pJ343) [12]. pSP64-HIF-1 was generated by excision of *hif-1* cDNA from pR33 [13] and insertion into pSP64 (Promega). EMSA reactions (20 µl) were performed in reaction buffer (10 mM Tris-HCl, pH 7.5, 4% glycerol, 1 mM MgCl_2_, 1 mM DTT) containing ^32^P-labeled wildtype or HRE3m probes (0.4–1 ng), poly dI-dC (80 ng) and HIF-1 and/or AHA-1 (1–4 µl) at room temperature for 30 min. *Ce*HIF-1 antibody (2 ul) was added during the last 5 min of the binding reactions. For competition experiments, unlabeled IDE or HRE3m DNA (10–100× molar amounts) was added to the reactions 5 min before addition of ^32^P-labeled probes. Samples were fractionated on a 5% non-denaturing polyacrylamide gel (37.5∶1 acrylamide∶bis).

### Metal content by inductively-coupled plasma-optical emission spectroscopy (ICP)

Synchronized L1 worms were obtained by treating gravid adults from each strain with alkaline hypochlorite followed by hatching eggs in S-basal medium overnight. L1 worms were grown on OP-50 seeded NGM plates. L4 worms were washed extensively with M9 buffer and incubated in M9 buffer for 30 min at room temperature to allow for purging of the gut followed by two rinses with ddH2O. Empty tubes were run in parallel to serve as controls. Samples were pelleted and frozen at −80C°. Samples and controls were brought up in 200 ul of metal free 40% nitric acid (Optima) and heated to 95°C for 2 min in a heating block. Solubilized samples were diluted to a final nitric acid concentration of 10% with ddH2O and measured on an Optima 3000 XL ICP-OES (Perkin Elmer). Serial dilutions of commercially available mixed metal standards were used to calibrate the instrument. Results were normalized to the simultaneously acquired signal for sulfur for each sample [63]. The data are presented as a percentage of N2 wildtype animals. At least three independent biological replicates were performed.

### Statistical analysis

Data are presented as the means ± SEM. Two-tailed unpaired Student's t test were used for statistical analysis. Data are considered statistically significant at *p*<0.05.

## Supporting Information

Figure S1 *ftn-1::GFP-his* and *smf-3(-1500)::GFP-his* expression are regulated by iron, but not by MnCl_2_. MnCl_2_ has been shown to regulate transcriptional expression of *smf-3* [28], [29]. To determine whether MnCl_2_ regulates expression of *smf-3(-1500)::GFP-his*, L1 stage worms were cultured on NGM, NGM-100 uM BP, NGM-100 uM BP plus 100 uM FAC and NGM-100 uM BP plus 100 µM MnCl_2_ for 18 hr. Worms were harvested and GFP expression was quantified by the COPAS Biosort. Dot plots of sorted worms are shown where each dot represents one worm. Mean GFP fluorescence (± SEM) is expressed as a percentage of worms grown on NGM (n = 1000 worms/treatment), *p<0.0001. (A) BP increases *smf-3(-1500)::GFP-his* expression, which is blocked by addition of equimolar amount of FAC plus BP. By contrast, GFP expression is not reduced when MnCl_2_ is present with BP. (B) BP reduces *ftn-1::GFP-his* expression and is reversed when FAC is present with BP. Addition of MnCl_2_ with BP did not increase GFP expression.
(EPS)

Figure S2 *smf-3(-250)::GFP-his* expression is regulated by HIF-1. GFP-his reporters were constructed containing 250 nt of *smf-3* promoter sequences 5′ to the initiator ATG (Figure 6A). This reporter contains the entire *smf-3* IDE. To determine whether *smf-3(-250)::GFP-his* was regulated by HIF-1, L1 stage worms carrying *smf-3(-250)::GFP-his* were spotted on *hif-1* or control (empty vector L4400) RNAi plates supplemented with or without 100 uM BP. GFP fluorescence was quantified by the COPAS Biosort after 18 h. Dot plots of sorted worms are shown where each dot represents one worm. Mean GFP fluorescence (± SEM) is reported as a percentage of control RNAi fed worms on NGM (n = 650 worms/treatment), *p<0.0001. BP increases GFP expression in control RNAi fed worms after growth on NGM-BP as compared to NGM-control fed RNAi. By contrast, BP-induced increased GFP expression is inhibited in worms fed *hif-1* RNAi.
(EPS)

Table S1 Sequences of primers used in this study.
(DOC)
