# Supplementary material for: HIF-1 Regulates Iron Homeostasis in Caenorhabditis elegans by Activation and Inhibition of Genes Involved in Iron Uptake and Storage
Source: PLoS Genet. 2011 Dec 15;7(12):e1002394. doi: 10.1371/journal.pgen.1002394 (PMC3240588; doi:10.1371/journal.pgen.1002394)
Supplement: Table S1 — Sequences of primers used in this study. (DOC) [file pgen.1002394.s003.doc]

| *ftn-1* (C5456.14) forward | 5'-GAATTCAAGCTGTCCGTGG-3' |
| --- | --- |
| *ftn-1* (C5456.14) reverse | 5'-CGAATGTACCTGCTCTTCC-3' |
| *ftn-2* (D1037.3) forward | 5'-GTTAACAAGCAGATCAACATT-3' |
| *ftn-2* (D1037.3) reverse | 5'-CATTCTCTGGCTTCTGGATG-3' |
| *smf-3* (Y69A2AR.4) forward | 5’ GATGTTGGTAGAGTCGGCG-3' |
| *smf-3* (Y69A2AR.4) reverse | 5'- CATCAGGAGCACTAACTCC-3' |
| *act-1* ((T04C12.6) forward | 5'- CCAAGAGAGGTATCCTTACC-3' |
| *act-1* ((T04C12.6) reverse | 5'-CTTGGATGGCGACATACATG-3' |
| *ftn-1* IDE (ChIP) forward | 5'-AACAGCTCACGTAGCCAATG-3' |
| *ftn-1* IDE (ChIP) reverse | 5'- TGTATGGTTGTCTCGGCATC-3' |
| *ftn-1* coding region (ChIP) forward | 5'- TATCGCACTTCGGAACATTG-3' |
| *ftn-1* coding region (ChIP) reverse | 5'- TCAAGGACAGTTCCCCACTC-3' |
| *smf-3* (1500) GFP construct forward | 5'- GTCGACATTTGAATTCAGCTCACCTAGAGC-3' |
| *smf-3* GFP construct reverse | 5'- GCTAGCGATTTCCTGACGAAATAGTATG-3' |
| *smf-3* (250) GFP construct forward | 5’-GTCGACCTTCTACGCACAAGCATCATACC-3’ |

**Sequences of primers used in this study**
